# Diffusion magnetic resonance imaging study of schizophrenia in the context of abnormal neurodevelopment using multiple site data in a Chinese Han population

**DOI:** 10.1038/tp.2015.202

**Published:** 2016-01-19

**Authors:** Y Li, S Xie, B Liu, M Song, Y Chen, P Li, L Lu, L Lv, H Wang, H Yan, J Yan, H Zhang, D Zhang, T Jiang

**Affiliations:** 1 Queensland Brain Institute, The University of Queensland, Brisbane, QLD, Australia; 2 Brainnetome Center, Institute of Automation, Chinese Academy of Sciences, Beijing, China; 3 National Laboratory of Pattern Recognition, Institute of Automation, Chinese Academy of Sciences, Beijing, China; 4 Department of Psychiatry, Xijing Hospital, The Fourth Military Medical University, Xi'an, China; 5 Peking University Sixth Hospital/Institute of Mental Health, Beijing, China; 6 Key Laboratory of Mental Health, Ministry of Health (Peking University), Beijing, China; 7 Department of Psychiatry, Henan Mental Hospital, The Second Affiliated Hospital of Xinxiang Medical University, Xinxiang, China; 8 Henan Key Lab of Biological Psychiatry, Xinxiang Medical University, Xinxiang, China; 9 Department of Psychology, Xinxiang Medical University, Xinxiang, China; 10 Center for Life Sciences/PKU-IDG/McGovern Institute for Brain Research, Peking University, Beijing, China; 11 Key Laboratory for NeuroInformation of Ministry of Education, School of Life Science and Technology, University of Electronic Science and Technology of China, Chengdu, China; 12 CAS Center for Excellence in Brain Science and Intelligence Technology, Institute of Automation, Chinese Academy of Sciences, Beijing, China

## Abstract

Schizophrenia has increasingly been considered a neurodevelopmental disorder, and the advancement of neuroimaging techniques and associated computational methods has enabled quantitative re-examination of this important theory on the pathogenesis of the disease. Inspired by previous findings from neonatal brains, we proposed that an increase in diffusion magnetic resonance imaging (dMRI) mean diffusivity (MD) should be observed in the cerebral cortex of schizophrenia patients compared with healthy controls, corresponding to lower tissue complexity and potentially a failure to reach cortical maturation. We tested this hypothesis using dMRI data from a Chinese Han population comprising patients from four different hospital sites. Utilizing data-driven methods based on the state-of-the-art tensor-based registration algorithm, significantly increased MD measurements were consistently observed in the cortex of schizophrenia patients across all four sites, despite differences in psychopathology, exposure to antipsychotic medication and scanners used for image acquisition. Specifically, we found increased MD in the limbic system of the schizophrenic brain, mainly involving the bilateral insular and prefrontal cortices. In light of the existing literature, we speculate that this may represent a neuroanatomical signature of the disorder, reflecting microstructural deficits due to developmental abnormalities. Our findings not only provide strong support to the abnormal neurodevelopment theory of schizophrenia, but also highlight an important neuroimaging endophenotype for monitoring the developmental trajectory of high-risk subjects of the disease, thereby facilitating early detection and prevention.

## Introduction

Over the past two decades, the advancement of neuroimaging techniques and associated computational methods has provided us with insight into the causes, progression and even treatment of schizophrenia,^1^ enabling quantitative re-examination of classical and popular theories on the pathogenesis of the disease.^2^ However, the majority of previous findings have exhibited relatively poor consistency and replication,^3^ likely due primarily to the relatively small sample size in each single study and the multitude of variables that can affect brain development.^4^ The aim of this study is to investigate the ‘abnormal neurodevelopment' theory of schizophrenia, using expertly collected and well-curated neuroimaging data.

In one recent perspective, it was forecasted that if researchers and clinicians approached schizophrenia as a neurodevelopmental disorder, prevention focusing on the pre-psychotic illness phase would be possible by 2030.^5^ From a developmental perspective, schizophrenia is viewed as a consequence of genetic predisposition and early adverse events, such as mid-gestational insults, that are latent throughout the first two decades of life and become evident as psychosis in early adulthood.^6^ This theory fits with the emerging consensus that adolescence as well as pre- and perinatal development are critical periods during which susceptibility genes and environmental factors may contribute to the future onset of schizophrenia. When considering schizophrenia as a deviation from normal neurodevelopment, a better understanding of normative brain development is essential to understand the processes underlying schizophrenia pathology.

Several groups have successfully related cortical microstructural changes during early neurodevelopment, such as increases in tissue complexity due to dendritic arborization and synapse formation, to measurements derived from diffusion magnetic resonance imaging (dMRI).^7, 8, 9^ It was proposed that increasing cellular density and complexity led to a decrease in tissue water content and thus a fall in mean diffusivity (MD) under dMRI examination, a finding that has been validated in neonatal piglets,^10^ cats^11^ and mice.^12^ Furthermore, a recent dMRI study of 65 human infants between 27 and 46 weeks post-conception showed that MD decreased consistently throughout the study period and was associated with a steady increase in cellular and synaptic complexity.^13^

Inspired by the above-mentioned dMRI findings, we propose that in the context of abnormal neurodevelopment in schizophrenia, MD in the cerebral cortex of schizophrenia patients should be increased relative to healthy controls, corresponding to decreased tissue complexity. This may reflect a failure to reach the final state of cortical maturation. We tested this hypothesis using dMRI data acquired from a Chinese Han population comprising patients from four different sites.

## Materials and methods

### Subjects

In the current study, 313 schizophrenia patients were recruited from 4 different hospitals in China: Peking University Sixth Hospital (Site 1); Beijing Huilongguan Hospital (Site 2); Xijing Hospital (Site 3) and Henan Mental Hospital (Site 4). For each site, all patients were assessed using the Structured Clinical Interview for DSM disorders (SCID), and diagnosed by experienced psychiatrists as meeting the criteria for schizophrenia per the Diagnostic and Statistical Manual for Mental Disorders 4th Edition text revision (DSM-IV-TR). The psychotic symptoms of patients were evaluated using the Positive and Negative Syndrome Scale (PANSS).^14^ Each patient group comprised a mixture of in- and outpatients, some of whom were reporting their first episode of schizophrenia. The patients' antipsychotic medication histories were not available when this study was performed. In total 307 healthy controls were recruited by advertisement from the same geographical areas as patients, with no current or past axis I or II disorders (DSM-IV-TR) as screened by the SCID-Non-Patient Version. Additional exclusion criteria for all subjects included current or past neurological illness, substance abuse or dependence, pregnancy, and prior electroconvulsive therapy or head injury resulting in loss of consciousness. All subjects were within the 18–45 age range, right-handed and Chinese Han. Ethical approval was granted from the relevant Ethics Committees, and written informed consent was obtained from all subjects. Detailed demographic and clinical characteristics of subjects from each site are shown in the Supplementary Table S1.

### MRI data acquisition and preprocessing

Diffusion-weighted images were acquired on a 3.0 T Siemens TrioTim Scanner (Siemens, Munich, Germany) (Sites 1, 2 & 3) or a 3.0 T Siemens Verio Scanner (Siemens) (Site 4) with an eight-channel SENSE head coil. To ensure equivalent and high quality data acquisition, the scanning protocols for all four sites were set up by the same experienced expert. Acquisition time for dMRI data of one subject was ~10 min, which was consistent across all four sites. The major acquisition parameters included: field-of-view=256 × 256 mm^2^, matrix size=128 × 128, slices=50 (with no gap), voxel size=2 × 2 × 3 mm^3^, repetition time (TR)=7000 ms (8400 ms for Site 4), echo time (TE)=92 ms (91 ms for Sites 3 & 4) and flip angle=90°. For each subject, a total of 65 images were acquired, including 1 non-diffusion-weighted image (*b*=0 s mm^−2^) and 64 diffusion-weighted images (I=1000 s mm^−2^) with 64 non-collinear gradient directions. Corrections for eddy current distortions and head motion were performed by aligning all diffusion-weighted images to the non-diffusion-weighted image (the b0 volume) using FSL (FSL 5.0; http://www.fmrib.ox.ac.uk/fsl).^15^ Subsequently, the b-matrix of each subject was reoriented accordingly to provide a more accurate estimate of tensor orientations. The brain mask in diffusion space was separated from the skull using the Brain Extraction Tool. The dMRI data of each individual were visually inspected by two specialists to make sure there were no apparent artifacts arising from acquisition or data processing procedures.

### Comprehensive analyses of cortical MD based on dMRI

#### VBA of MD utilizing tensor-based registration

We performed two-sample *t-*tests in a voxel-based manner to test our hypothesized differences in cortical MD measurements between schizophrenia patients and healthy controls. As in all voxel-based analysis (VBA) approaches, accurate cross-subject alignment of anatomically related regions is of paramount importance and the limitation of using scalar image (such as MD map itself) based registration, which may considerably affect the reliability of results, has been detailed recently.^16^ Therefore, we replaced scalar image-based registration by full tensor information registration, realized by DTI-TK,^17, 18^ which was the overall winner of a registration algorithm challenge^19^ and is proposed to enhance the accuracy of VBA.^16^

In detail, the native diffusion tensor image of each individual was first calculated using FSL and then normalized, using rigid, affine and deformable alignment provided by DTI-TK (http://dti-tk.sourceforge.net), to the IIT Human Brain Template,^20^ which was recently developed by high angular resolution diffusion imaging of 72 healthy human subjects (42 females: 26.6±4.8 years of age, 20–39 years age range; 30 males: 31.9±4.9 years of age, 22–40 years age range). The normalized diffusion tensor images of all subjects (both schizophrenia patients and healthy controls from the same site) were aligned in the ICBM-152 space,^21^ with a spatial resolution of 1 mm isotropic. An MD image of each individual was then calculated from his/her normalized diffusion tensor volume, rather than computed from the individual's native diffusion tensor image to better account for tensor interpolation due to registration. The resultant MD image, which was anatomically aligned across subjects within standard ICBM-152 space with 1 mm isotropic resolution, was then smoothed by a 4-mm full-width at half-maximum Gaussian kernel to reduce the effect of mis-registration in spatial normalization.^22^

Finally, we performed a two-sample *t*-test on the normalized MD images between schizophrenia patients and healthy controls in a voxel-based manner, using the Threshold-Free Cluster Enhancement (TFCE) method, which can enhance cluster-like structures without having to define an initial cluster-forming threshold or carry out a large amount of data smoothing.^23^ In addition, the voxel-wise statistical test was restricted to brain gray matter only by employing the gray matter tissue mask provided by the IIT Human Brain Template.^24^ Statistical significance was defined as *P*<0.05 (corrected for family-wise error corrected), after controlling for age and gender as variables of no interest. The above-described VBA of MD was carried out for each of the four sites independently, which can be thought of as a series of replication studies.

#### ROI analysis

To better clarify our findings from VBA, we further selected region-of-interests (ROIs) that consistently showed significantly different MD between schizophrenia patients and controls across all four sites, and subsequently calculated the average MD value of each selected ROI for statistical analyses.

In detail, we employed the Desikan atlas provided by the IIT Human Brain Template for anatomical reference,^24^ which segmented the brain gray matter into 86 gyral-based neuroanatomical regions (including 34 cortical ROIs and 9 sub-cortical ROIs in each hemisphere). For each site, results from VBA were clusters of voxels with significantly different MD within brain gray matter between schizophrenia patients and healthy controls, which were in the standard ICBM-152 space. By overlapping with the Desikan atlas, we not only identified which cortical or sub-cortical ROI showed significantly different MD in schizophrenia patients consistently across all four sites, but also how many voxels found by the VBA were located in that ROI, indicating how much the corresponding ROI was associated with the disease compared with other brain regions. Utilizing the inverse transformation previously obtained from tensor-based registration, we warped the identified ROIs from MNI152 space back into the native dMRI space of each individual, and the average MD of each ROI was then calculated for statistical analyses. The following should be noted: (1) the mean MD utilized here for ROI analysis was averaged across the entire ROI, rather than only across those voxels identified by VBA as significant, to provide a characteristic of the neuroanatomical region that is independent of VBA findings across different sites; (2) the MD utilized here for ROI analysis was calculated based on the native diffusion tensor of each individual (with acquired resolution 2 × 2 × 3 mm^3^), which was different to the MD calculated based on the normalized diffusion tensor in VBA (with registered resolution 1 mm isotropic). This provided validation for our analyses by investigating the same data using different methods with different protocols.

General linear model (GLM) analyses were performed using SPSS (IBM SPSS Statistics, Version 19; IBM, Armonk, NY, USA), with average MD of ROIs being dependent variables, group factor (two groups: schizophrenia patients or healthy controls) being an independent variable, and age, gender and whole brain gray matter volume (estimated based on the brain gray matter tissue mask warped from standard space into native dMRI space of the individual) being variables of no interest. The GLM analyses were carried out independently for each of the four sites. The significance of the result was determined using a threshold of *P*<0.05 (corrected for multiple tests across sites and ROIs).

In addition, by pooling schizophrenia patients from all four sites, partial correlation analyses between the average MD of ROIs and the clinical measurements, including the PANSS positive and PANSS negative scores, were performed with age, gender, whole brain gray matter volume and the factor of different sites as variables of no interest. Statistical significance was set at *P*<0.05 (corrected for multiple tests across different ROIs).

## Results

**Figure 1:**
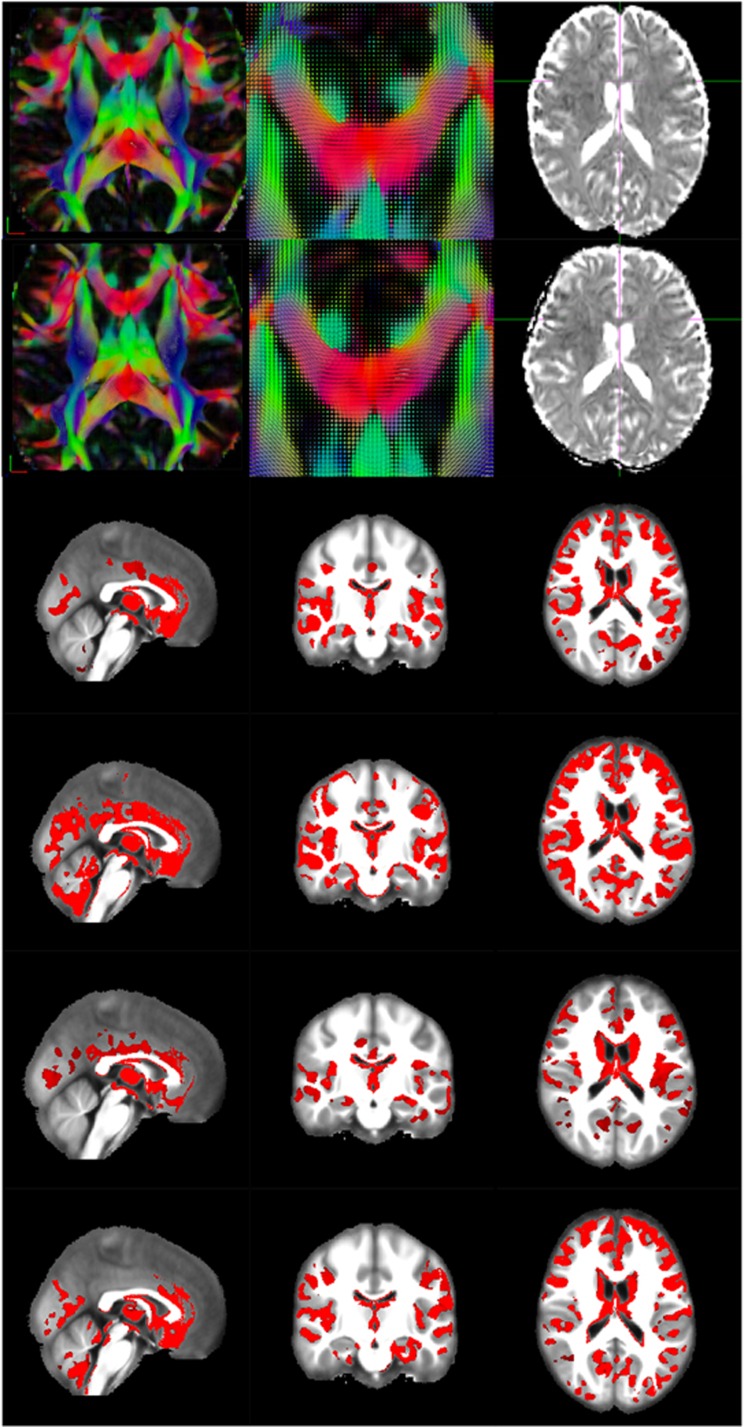
Significantly increased MD found by VBA in brain gray matter of schizophrenia patients. Rows 1 and 2 show data from one healthy subject and one schizophrenia patient randomly selected from the same site. The left column shows an axial view of the normalized tensor image, which was color-coded according to the orientation of the eigenvector of the tensor (red: left–right, green: anterior-posterior and blue: superior-inferior) and was set to the same slice within the ICBM-152 standard space across two subjects. The right column shows an axial view of the MD map calculated from the normalized tensor of the subject, which was set to the same slice as shown in the left column. The middle column shows the zoomed-in view of the normalized tensor, which was centered at the genu of corpus callosum across two subjects, indicated by the cross in the right column. Rows 3–6 show the VBA results, each row representing the results from one site. Brain regions with significantly increased MD (red color) in schizophrenia patients compared with healthy controls were overlaid on the MNI152 image (background) for presentation. Each column in the figure represents different views of the same result (left: sagittal, middle: coronal and right: axial), and shows the same slices within the MNI152 image across sites. ICBM, The International Consortium for Brain Mapping; MD, mean diffusivity; VBA, voxel-based analysis.

As shown in Figure 1, accurate alignment of anatomically related regions was achieved across subjects by tensor-based registration using DTI-TK. In addition, for every hospital site VBA revealed significantly increased MD in the gray matter of schizophrenia patients as compared with healthy controls, despite differences in psychopathology, exposure to antipsychotic medication and the scanners used for image acquisition (see Figure 1). No significant decrease in MD was observed in schizophrenia patients from any site as compared with healthy controls.

**Figure 2:**
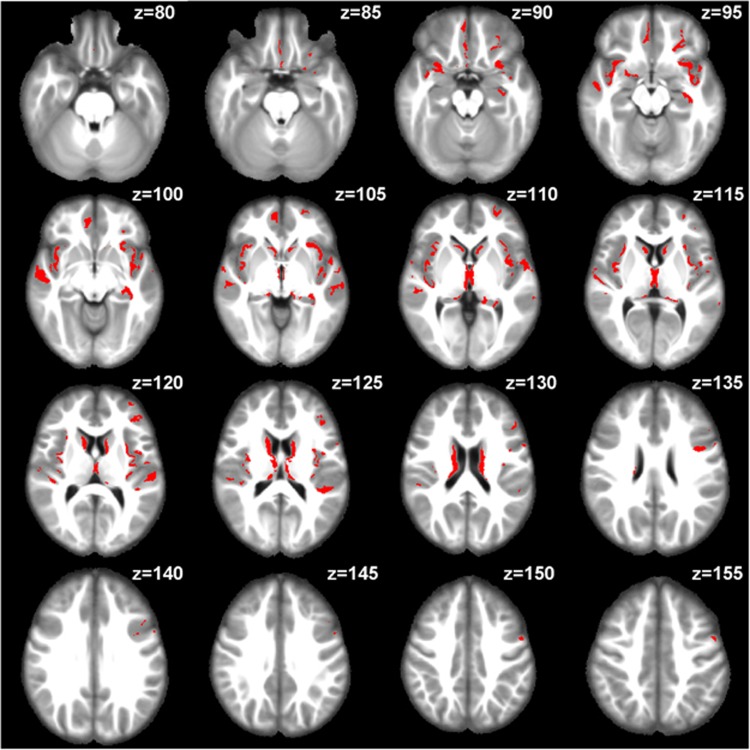
Clusters of voxels with significantly increased MD in schizophrenia patients. We included 16 axial slices, starting from *z*=80 to *z*=155 with a 5-slice gap in MNI152 standard space (1mm isotropic resolution), revealing clusters of voxels (red color) identified by VBA as having significantly increased MD in schizophrenia patients consistently across all four sites. MD, mean diffusivity; VBA, voxel-based analysis.

**Figure 3:**
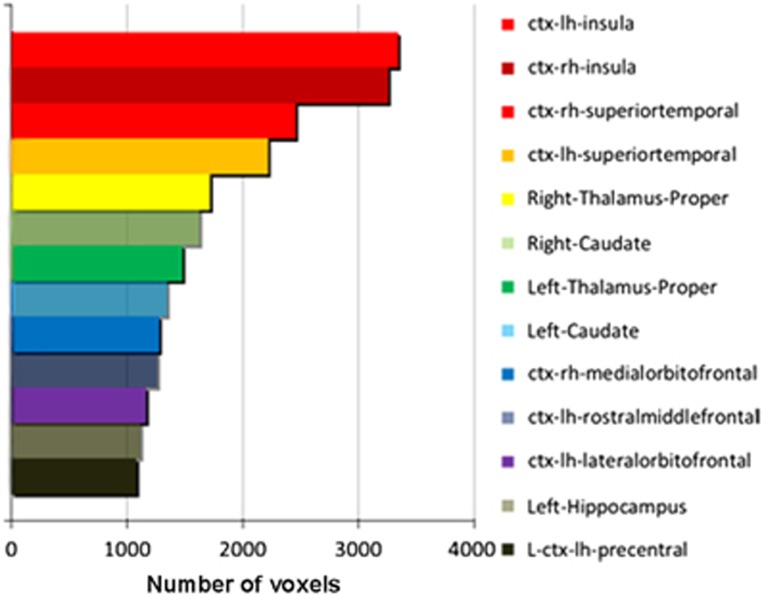
The 13 ROIs with significantly increased MD in schizophrenia patients. The ROIs were ordered by the number of voxels (1 mm isotropic resolution in MNI152 standard space) they contained with significant differences between patients and controls, as assessed by VBA and which held across all four sites. The name of each ROI is shown on the right; ROI names are as defined by the Desikan atlas. MD, mean diffusivity; ROI, region-of-interest; VBA, voxel-based analysis.

By pooling the VBA results from each site, we further identified clusters of voxels throughout the gray matter of schizophrenia patients with significantly increased MD, a finding that was consistent across all four sites (see Figure 2). According to the Desikan atlas,^24^ these clusters of voxels involved a total of 13 neuroanatomical ROIs, each of which included more than 1000 voxels identified by VBA as significant (1 mm isotropic resolution in the MNI152 standard space), with the biggest clusters located in the bilateral insular cortex (see Figure 3). Other ROIs included bilateral superior temporal cortex, bilateral thalamus, bilateral caudate, right medial orbitofrontal cortex, left rostral middle frontal cortex, left lateral orbitofrontal cortex, left precentral cortex and left hippocampus, the names of which were defined by the Desikan atlas.^24^

**Figure 4:**
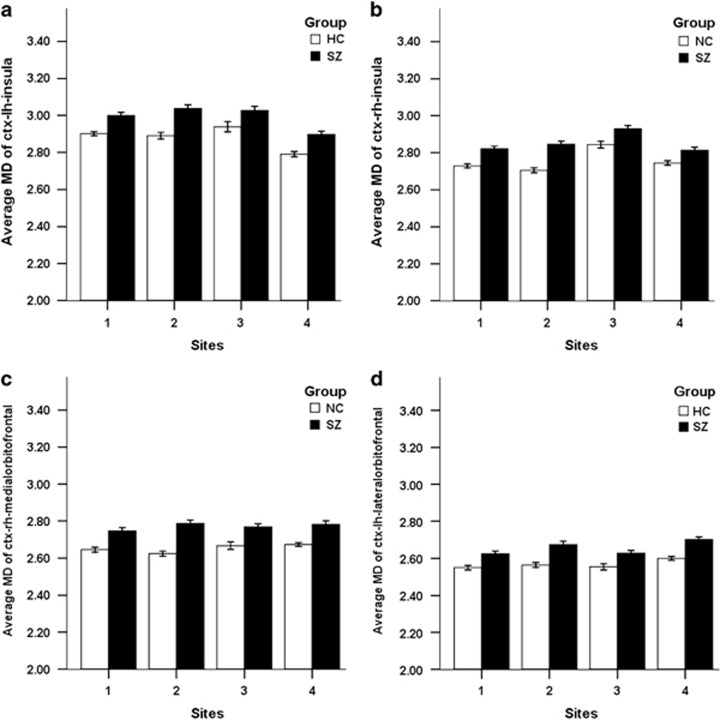
The four ROIs with significantly increased MD in schizophrenia patients as determined by GLM analysis. Average MD of ctx-lh-insula (**a**), ctx-rh-insula (**b**), ctx-rh-medialorbitofrontal (**c**) and ctx-lh-lateralorbitofrontal (**d**). The name of each ROI was defined by the Desikan atlas. Error bars represent s.e. GLM, general linear model; HC, healthy controls; MD, mean diffusivity; ROI, region-of-interest; SZ, schizophrenia patients.

**Figure 5:**
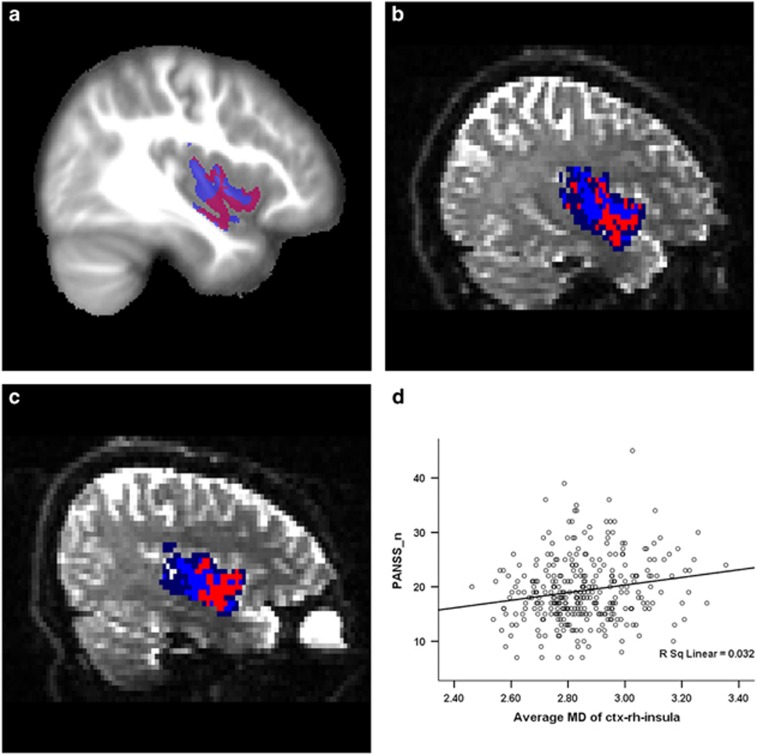
Significant correlation between average MD of right insular cortex and PANSS negative score. (**a**) Representative sagittal slice within the MNI152 standard space, in which the MNI152 image was used as background and the right insular cortex from the Desikan atlas was overlaid (blue). The cluster of voxels found by VBA with significantly increased MD across all four sites is shown in red. Similar to **a**, representative sagittal slice is shown for one healthy subject (**b**) and one schizophrenia patient (**c**) randomly selected from the same site, in which the non-diffusion-weighted image was used as a background, and the right insular cortex from the Desikan atlas (blue) and the cluster of voxels found by VBA (red color) were warped onto individual dMRI space and overlaid for presentation. (**d**) Correlation between average MD of right insula and PANSS negative score (PANSS_n) for all patients across all four sites. MD, mean diffusivity; PANSS, Positive and Negative Syndrome Scale; VBA, voxel-based analysis.

GLM analyses of the 13 identified ROIs, which were performed on the average MD of the entire ROI after being warped back into each individual's native dMRI space, were carried out for each site with age, gender and whole brain gray matter volume controlled for as variables of no interest. Results for each ROI were considered significant only when the raw *P*-value of group effect (schizophrenia patients or healthy controls) was lower than 0.05/(13 × 4)=9.615 × 10^-4^ for all four sites. Four out of the 13 ROIs, including bilateral insula, right medial orbitofrontal cortex and left lateral orbitofrontal cortex, survived the relatively stringent criteria mentioned above (Figure 4), with schizophrenia patients showing significantly increased MD compared with healthy controls (see Supplementary Table S2). In addition, partial correlation analyses were performed between the average MD of these four ROIs and clinical measurements, including PANSS_p and PANSS_n scores, with age, gender, whole brain gray matter volume and the factor of different sites controlled for as variables of no interest. As shown in Figure 5, a significant positive correlation (Pearson's correlation coefficient=0.175 and *P*-value=0.002) was found between the average MD of right insular cortex and the PANSS negative score (results of other regions can be found in Supplementary Table S3).

Finally, considering the patients' antipsychotic medication histories were not available, we further analyzed our findings to increase the integrity of our study. In detail, we further separated the schizophrenia patients of each site into two groups: (1) patients reporting their first episode of schizophrenia with illness duration no more than 12 months, which represented patients with minimum exposure to any antipsychotic medication; and (2) the rest of the patients from the same site, which represented patients with relatively complex clinical background. Demographic details of these two groups of patients across four sites can be found in Supplementary Table S4. Then, focusing on the average MD of right insular cortex, we re-performed GLM analyses and partial correlation analyses as described in previous sections for each of these two patient groups independently. The results were consistent with our findings above (see Supplementary Table S5), except that a significant correlation between the average MD of the right insula and the PANSS_n score was no longer observed in patients who were reporting their first episode and had an illness duration no more than 12 months. We believe this is probably due to the relatively small sample size after separating the patients. Although the lack of antipsychotic medication histories prevented us from performing a restricted control of drug effect in the current study, the fact that we observed well-consistent results despite different groupings of schizophrenia patients across four independent sites strongly indicates that the increased MD we observed in schizophrenia patients reflects abnormal neurodevelopment, regardless of exposure to antipsychotic medication.

## Discussion

Using dMRI of schizophrenia patients located at four different hospital sites, we observed significantly increased MD in the cortex compared with healthy controls, providing strong support to our hypothesis that schizophrenia patients exhibit an immature cortex resulting from abnormal neurodevelopment. More specifically, patients showed increased MD in the bilateral insular and prefrontal cortices, identified by both VBA and ROI analysis. In addition, the average MD of the right insular cortex showed a significant positive correlation with clinical measurements across all schizophrenia patients: a higher MD of the right insular cortex, indicating less tissue complexity, was associated with a higher PANSS negative score, indicating more serious psychotic symptoms.

One of the strengths of our investigation is that high quality dMRI data, with high spatial and angular resolution, was acquired using a common protocol across different sites from unique samples of schizophrenia patients with the same ethnic origin. Our analyses on these expertly collected and curated neuroimaging data were performed for each site independently, meaning they can be thought of as a series of replication studies. Increasing attention has been paid to the problem of running small studies with low statistical power in biomedical research.^4, 25, 26^ To combat the problem, invaluable efforts have been made using traditional meta-analyses to pool statistical findings reported in the literature, identifying considerable heterogeneity in the effect sizes and patterns of brain differences related to schizophrenia, particularly for studies using structural MRI to identify brain morphological abnormalities.^27, 28, 29, 30, 31, 32, 33, 34^ More recently, exciting advances have been made through national and international collaborations that have allowed thousands of schizophrenia brains from multiple sites to be analyzed using multi-modal MRI.^3, 35, 36, 37, 38, 39, 40, 41, 42, 43, 44, 45, 46, 47, 48, 49, 50, 51, 52, 53, 54, 55, 56, 57, 58^ However, the majority of previous multi-site studies focused on pooling data across sites using standardization and calibration methods, which lack well-accepted standards, particularly for dMRI data. By adopting a completely different data analysis strategy, our current investigation can be thought of as a series of high quality replication studies across multiple sites. A further strength of our study is that our findings were obtained by a completely data-driven method of VBA, based on the state-of-the-art tensor-based registration algorithm, despite differences in psychopathology, exposure to antipsychotic medication and the scanners used for image acquisition across patients from different sites. In particular, the majority of brain structural abnormalities we identified occurred within the bilateral insular cortex, and the average MD of the right insula was significantly correlated with the degree of illness across patients. Remarkably, using T1-weighted MRI, a recent neuroimaging study of schizophrenia across four ethnically distinct cohorts (white Caucasians, African-Caribbean, Japanese and Chinese) reported reduced gray matter volume only in the right insula of patients, which was consistently found for each cohort.^59^ It is encouraging that findings from our current study are convergent with this previous independent multi-site study, and together they provide strong evidence that a neuroanatomical signature of schizophrenia—regardless of demographic variations, incidence and clinical expression of patients, and MRI methods—may partly reside in the insular cortex and present itself as decreased tissue complexity in the context of abnormal neurodevelopment.

Although proposed more than two decades ago,^60, 61, 62^ evidence for and recognition of the implications of shifting to a neurodevelopmental approach in schizophrenia were not available until recently.^63, 64^ The model arising from this neurodevelopmental perspective consists of an early insult (genetic and environmental factors), a latent period through much of neural development, and the emergence of psychosis in late adolescence or early adulthood.^5^ Two possibilities have been put forward in light of the model. One is that a lesion early in development does not manifest until a much later developmental stage, when compensatory changes can no longer suffice.^65^ The other is that the developmental lesion influences a pathway or regulatory process, such as the fine tuning of excitatory and inhibitory synapses in the prefrontal cortex, which may have only subtle effects until a precise balance is required in late adolescence.^5^ Either way, the neurodevelopmental perspective of schizophrenia implies a failure to reach the final state of cortical maturation, resulting in the retention of an immature cortex.^6^ Our current findings of increased MD in the insula and prefrontal cortex of schizophrenia patients may be the result of a developmental lesion as determined by macroscopic neuroimaging examination. The insular cortex has been implicated in self-awareness,^66^ emotional regulation^67^ and the processing of salience,^68^ which are known to be affected in schizophrenia,^69^ and a reduced insular cortex volume is one of the most commonly identified regional structural effects in schizophrenia.^70, 71^ The prefrontal cortex is also consistently implicated in the pathophysiology of schizophrenia, the dysfunction of which is thought to be an underlying substrate for the disorder.^72^ Our VBA investigations also identified, across all hospital sites, increased MD within the thalamus, hippocampus and temporal cortex, all of which form part of the limbic system, as do the insular and prefrontal cortices. Abnormal anatomical connections between these brain regions have been shown to be associated with the psychiatric and cognitive manifestations of schizophrenia.^73^ Importantly, our findings of increased MD in the insula and prefrontal cortex of schizophrenia patients remained valid when controlling for whole brain gray matter volume as a variable of no interest. Volume reduction in the insular cortex due to neuron loss is one of the most consistent neuropathological findings of schizophrenia to date,^74^ and our observation implies that the diffusion of water molecules in patients was less hindered, likely due to reduced dendritic and/or axonal arborization in the absence of any neuron loss.

In conclusion, our investigation provides evidence for immature cortical development in the limbic system of the schizophrenic brain, mainly involving the bilateral insular and prefrontal cortices. Our findings were drawn from expertly collected multi-site dMRI data from a Chinese Han population and were true despite differences in the patients' psychopathology, exposure to antipsychotic medication and the scanners used for image acquisition. In light of the existing literature, we speculate that the increased MD we observed in schizophrenia patients may represent a neuroanatomical signature of the disorder, reflecting microstructural deficits that result from a developmental lesion. Our findings not only provide strong support to the abnormal neurodevelopment theory of schizophrenia, but also highlight an important neuroimaging endophenotype for monitoring the developmental trajectory of high-risk subjects of the disease, thereby facilitating early detection and prevention.
