# Supplementary material for: Diffusion magnetic resonance imaging study of schizophrenia in the context of abnormal neurodevelopment using multiple site data in a Chinese Han population
Source: Transl Psychiatry. 2016 Jan 19;6(1):e715–. doi: 10.1038/tp.2015.202 (PMC5068876; doi:10.1038/tp.2015.202)
Supplement: Supplementary Information [file tp2015202x1.doc]

Supplementary Information for

**Diffusion Magnetic Resonance Imaging Study of Schizophrenia in the Context of Abnormal Neurodevelopment Using Multiple Site Data in a Chinese Han Population**

**Authors**: Yonghui Li, PhD^1, #^, Sangma Xie, BS^2, 3, #^, Bing Liu, PhD^2, 3^, Ming Song, PhD^2, 3^, Yunchun Chen, MD^4^, Peng Li, PhD^5, 6^, Lin Lu, PhD^5, 6^, Luxian Lv, PhD^7, 8^, Huaning Wang, MD^4^, Hao Yan, PhD^5, 6^, Jun Yan, MD^5, 6^, Hongxing Zhang, MD^7, 9^, Dai Zhang, MD, PhD^5, 6, 10^, and Tianzi Jiang, PhD^1, 2, 3, 11, 12, *^

^1^ Queensland Brain Institute, The University of Queensland, Brisbane, QLD 4072, Australia

^2^ Brainnetome Center, Institute of Automation, Chinese Academy of Sciences, Beijing 100190, China

^3^ National Laboratory of Pattern Recognition, Institute of Automation, Chinese Academy of Sciences, Beijing 100190, China

^4^ Department of Psychiatry, Xijing Hospital, The Fourth Military Medical University, Xi’an 710032, China

^5^ Peking University Sixth Hospital / Institute of Mental Health, Beijing 100191, China

^6^ Key Laboratory of Mental Health, Ministry of Health (Peking University), Beijing 100191, China

^7^ Department of Psychiatry, Henan Mental Hospital, The Second Affiliated Hospital of Xinxiang Medical University, Xinxiang 453002, China

^8^ Henan Key Lab of Biological Psychiatry, Xinxiang Medical University, Xinxiang 453002, China

^9^ Department of Psychology, Xinxiang Medical University, Xinxiang 453002, China

^10^ Center for Life Sciences / PKU-IDG / McGovern Institute for Brain Research, Peking University, Beijing 100871, China

^11^ Key Laboratory for NeuroInformation of Ministry of Education, School of Life Science and Technology, University of Electronic Science and Technology of China, Chengdu 610054, China

^12^ CAS Center for Excellence in Brain Science and Intelligence Technology, Institute of Automation, Chinese Academy of Sciences, Beijing 100190, China

^#^ Yonghui Li and Sangma Xie contributed equally to this work and should be considered as co-first authors.

^*^**Correspondence to**:

Tianzi Jiang, National Laboratory of Pattern Recognition, Institute of Automation, Chinese Academy of Sciences, Beijing 100190, China

Phone: +86 10 8254478

**Email:** jiangtz@nlpr.ia.ac.cn

**Tables:**

Table S1. Demographic and clinical details of the subjects

|  | Schizophrenia patients  (n=313) | | | | Healthy controls  (n=307) | | | |
| --- | --- | --- | --- | --- | --- | --- | --- | --- |
|  | Site-1  (n=90) | Site-2  (n=76) | Site-3  (n=77) | Site-4  (n=70) | Site-1  (n=100) | Site-2  (n=61) | Site-3  (n=48) | Site-4  (n=98) |
| Age | 26.66 (±6.18) | 28.73 (±7.58) | 26.31 (±6.42) | 26.88 (±5.88) | 25.71 (±5.37) | 26.22 (±5.97) | 32.12 (±7.00) | 29.83 (±7.07) |
| Gender | 56 M  34 F | 29 M  47 F | 42 M  35 F | 30 M  40 F | 53 M  47 F | 32 M  29 F | 28 M  20 F | 50 M  48 F |
| Years of education | 13.20 (±3.38) | 12.73 (±3.04) | 9.79 (±4.71) | 11.04 (±2.80) | 13.66 (±3.35) | 13.50 (±4.49) | 12.38 (±3.33) | 13.71 (±2.88) |
| Months of Illness duration | 51.14 (±51.47) | 63.64 (±57.86) | 24.60 (±29.31) | 40.96 (±41.40) | N/A | N/A | N/A | N/A |
| PANSS  positive | 23.57 (±4.65) | 26.22 (±3.28) | 23.03 (±4.98) | 22.29 (±2.79) | N/A | N/A | N/A | N/A |
| PANSS negative | 18.12 (±5.99) | 16.53 (±3.24) | 23.29 (±6.59) | 19.53 (±4.68) | N/A | N/A | N/A | N/A |

For age, years of education, months of illness duration, PANSS positive score and PANSS negative score, the data is organized in the format of mean (±standard error). For gender, the data is reported as number of males (M) and number of females (F). For schizophrenia patients from each site, some of the patients reported experiencing their first episode of schizophrenia (illness duration = 0 month), which led to a relatively large standard error for the measurement across all patients at that site.

Table S2. GLM analyses of average MD of ROIs identified by VBA

| ROI | Site | Raw *P*-value from GLM | | | |
| --- | --- | --- | --- | --- | --- |
|  |  | ***Group***  ***(Schizophrenia/Controls)*** | Age | Gender | GMV |
| ctx-lh-insula | 1 | ***5.606×10^-6^*** | 0.909 | 0.046 | 0.392 |
|  | 2 | ***2.401×10^-6^*** | 0.838 | 0.102 | 0.320 |
|  | 3 | ***3.197×10^-4^*** | 1.631×10^-4^ | 0.634 | 0.143 |
|  | 4 | ***1.238×10^-7^*** | 0.005 | 0.859 | 0.229 |
| ctx-rh-insula | 1 | ***4.155×10^-7^*** | 0.460 | 0.004 | 0.017 |
|  | 2 | ***6.556×10^-8^*** | 0.424 | 0.371 | 0.471 |
|  | 3 | ***2.227×10^-5^*** | 0.001 | 0.587 | 0.160 |
|  | 4 | ***2.285×10^-5^*** | 0.048 | 0.013 | 0.265 |
| ctx-rh-medialorbitofrontal | 1 | ***1.011×10^-5^*** | 0.291 | 0.002 | 0.002 |
|  | 2 | ***2.320×10^-8^*** | 0.269 | 0.002 | 0.003 |
|  | 3 | ***2.516×10^-4^*** | 0.292 | 0.678 | 0.858 |
|  | 4 | ***2.419×10^-7^*** | 0.218 | 0.054 | 0.829 |
| ctx-lh-lateralorbitofrontal | 1 | ***3.986×10^-5^*** | 0.061 | 0.018 | 0.003 |
|  | 2 | ***3.572×10^-4^*** | 0.688 | 0.290 | 0.079 |
|  | 3 | ***1.469×10^-4^*** | 0.015 | 0.978 | 0.738 |
|  | 4 | ***1.310×10^-9^*** | 0.015 | 0.196 | 0.798 |

GMV: gray matter volume.

Table S3. Partial correlation analyses between average MD of ROIs and clinical measurements.

| ROI | PANSS_p | PANSS_n |
| --- | --- | --- |
| ctx-lh-insula | 0.025 (0.650) | 0.106 (0.055) |
| ctx-rh-insula | -0.048 (0.385) | ***0.175 (0.002)*** |
| ctx-rh-medialorbitofrontal | -0.027 (0.627) | 0.028 (0.618) |
| ctx-lh-lateralorbitofrontal | 0.008 (0.887) | 0.032 (0.567) |

All data are shown in the form of Correlation Coefficient (*P*-Value).

Table S4. Demographic and clinical details of schizophrenia patients of two different groups.

|  | Schizophrenia patients  (total n=313) | | | | | | | | |
| --- | --- | --- | --- | --- | --- | --- | --- | --- | --- |
|  | Site-1  (total n=90) | | Site-2  (total n=76) | | Site-3  (total n=77) | | | Site-4  (total n=70) | |
|  | Group 1  (n=24) | Group 2  (n=66) | Group 1  (n=10) | Group 2  (n=66) | Group 1  (n=36) | Group 2  (n=41) | | Group 1  (n=20) | Group 2  (n=50) |
| Age | 24.36 (±5.47) | 27.49 (±6.25) | 27.70 (±7.77) | 28.88 (±7.60) | 24.89 (±6.03) | 27.11 (±5.99) | 25.25 (±4.74) | | 27.54 (±5.65) |
| Gender | 15 M  9 F | 41 M  25 F | 4 M  6 F | 25 M  41 F | 21 M  15 F | 21 M  20 F | 7 M  13 F | | 23 M  27 F |
| Years of education | 12.13 (±4.41) | 13.59 (±2.86) | 13.70 (±2.98) | 12.58 (±3.05) | 9.69 (±5.03) | 9.98 (±4.49) | 11.20 (±3.41) | | 10.98 (±2.55) |
| Months of Illness duration | 4.71 (±3.07) | 68.03 (±50.39) | 2.90 (±2.96) | 72.85 (±56.64) | 3.89 (±3.39) | 43.70 (±29.74) | 3.95 (±3.36) | | 55.76 (±40.32) |
| PANSS  positive | 23.96 (±4.51) | 23.42 (±4.73) | 28.70 (±4.27) | 25.85 (±2.96) | 24.17 (±4.25) | 21.93 (±5.43) | 21.70 (±3.29) | | 22.52 (±2.56) |
| PANSS negative | 17.67 (±6.41) | 18.29 (±5.87) | 15.10 (±1.79) | 16.74 (±3.37) | 22.72 (±5.76) | 23.78 (±7.36) | 20.10 (±4.89) | | 19.30 (±4.62) |

Group 1: patients reporting their first episode of schizophrenia with illness duration no more than 12 months.

Group 2: the rest of the patients from the same site.

For age, years of education, months of illness duration, PANSS positive score and PANSS negative score, the data is organized in the format of mean (±standard error). For gender, the data is reported as number of males (M) and number of females (F).

Table S5. GLM and partial correlation analyses of average MD of right insula cortex.

|  | Site | Group effect from GLM  (Schizophrenia/Controls) | Partial correlation with PANSS_n |
| --- | --- | --- | --- |
| Group 1 | 1 | 0.0004 | 0.113 (0.302) |
|  | 2 | 0.012 |  |
|  | 3 | 0.004 |  |
|  | 4 | 0.001 |  |
| Group 2 | 1 | 1.350×10^-5^ | 0.149 (0.028) |
|  | 2 | 3.028×10^-7^ |  |
|  | 3 | 3.853×10^-4^ |  |
|  | 4 | 1.147×10^-4^ |  |

Group 1: patients reporting their first episode of schizophrenia with illness duration no more than 12 months.

Group 2: the rest of the patients from the same site.

GLM analyses were carried out for each site with age, gender and whole brain gray matter volume controlled for as variables of no interest; the data shows the Raw *P*-value.

Partial correlation analyses were performed by pooling all schizophrenia patients from all 4 sites together, with age, gender, whole brain gray matter volume and the factor of different sites controlled for as variables of no interest; all data are shown in the form of Correlation Coefficient (*P*-Value).
